# Pulmonary vascular and airway changes in previously hospitalised COVID-19 patients: Long-term functional respiratory imaging findings correlate with reduced DLCO

**DOI:** 10.1371/journal.pone.0335075

**Published:** 2025-12-02

**Authors:** Ann Mari Svensson, Mikael Björnson, Magnus Sköld, Hans-Ulrich Kauczor, Malin Nygren-Bonnier, Judith Bruchfeld, Michael Runold, Anna Kistner, Sven Nyrén

**Affiliations:** 1 Department of Radiology, Karolinska University Hospital, Stockholm, Sweden; 2 Department of Molecular Medicine and Surgery, Karolinska Institutet, Stockholm, Sweden; 3 Department of Medicine, Solna, Karolinska Institutet, Stockholm, Sweden; 4 Department of Respiratory Medicine and Allergy, Karolinska University Hospital, Stockholm, Sweden; 5 Division of Immunology and Respiratory Medicine, Department of Medicine Solna and Center for Molecular Medicine, Karolinska Institutet, Stockholm, Sweden; 6 Department of Diagnostic and Interventional Radiology, University Hospital of Heidelberg, Heidelberg, Germany; 7 Translational Lung Research Center, Heidelberg, member of the German Center of Lung Research, Heidelberg, Germany; 8 Women’s Health and Allied Health Professionals Theme, Medical Unit Allied Health Professionals, Karolinska University Hospital, Stockholm, Sweden; 9 Division of Physiotherapy, Department of Neurobiology, Care Sciences and Society, Karolinska Institutet, Stockholm, Sweden; 10 Department of Infectious Diseases, Karolinska University Hospital, Stockholm, Sweden; 11 Department of Nuclear Medicine and Medical Physics, Karolinska University Hospital, Stockholm, Sweden; Kurume University School of Medicine: Kurume Daigaku Igakubu Daigakuin Igaku Kenkyuka, JAPAN

## Abstract

**Background:**

Persistent respiratory symptoms in COVID-19 patients have raised concerns about structural remodelling in the lung. We assessed structural changes and their correlation with reduced DLCO, eight months after discharge, in previously hospitalised COVID-19 patients.

**Materials and Methods:**

An exploratory observational study was conducted on 26 male patients (mean age: 60 years, range: 50–69) previously hospitalised for COVID-19. CT scans, performed eight months post-discharge, were analysed using functional respiratory imaging (FRI) to assess lung structure and function. Analyses were made based on diffusion capacity for carbon monoxide (DLCO).

**Results:**

Patients with low DLCO (≤75%; n = 9) exhibited a significantly lower proportion of small blood vessels with a cross-sectional area < 5 mm² compared to patients with normal DLCO (>75%; n = 17) (median (IQR) 56 (51–59) % vs. 60 (56–64) %, p = 0.008), as well as a reduced absolute volume of small vessels with a cross-sectional area < 5 mm² (129 (121–151) ml vs. 155 (132–175) ml, p = 0.025). Bronchial dilatation was more evident in the low DLCO group, with a higher ratio of airway volume to lobar volume (siVaw) (149 (138–165) % vs. 117 (93–132) %, p = 0.002). SiVaw showed a significant inverse relationship with DLCO (r = –0.56, p = 0.004, R² = 0.31). Lobar volumes were reduced in both DLCO groups, and more pronounced in the low DLCO group (72 (65–81) % vs. 90 (79–95) %, p = 0.001), as was total lung capacity (TLC) (73 (64–85) % vs. 92 (85–98) % of predicted, p = 0.003). FEV₁/FVC ratios were elevated in both groups, with a potential difference observed between the low and normal DLCO groups (110 (103–118) % vs. 105 (95–113) %, p = 0.074).

**Conclusions:**

We demonstrate long-term vascular and airway remodelling detected with FRI in previously hospitalised COVID-19 patients and highlight potential mechanisms underlying persistent pulmonary dysfunction and emphasise the need to investigate the underlying pathophysiology to identify potential individualised treatment strategies for this patient group.

## Introduction

Following the COVID-19 pandemic, a significant subset of patients exhibits persistent long-term impairment from multiple organs, including the lungs [1–3]. Respiratory symptoms, such as dyspnoea, were among the most common symptoms during the acute phase of COVID-19 and have also been identified as the second most prevalent long-term complication of COVID-19, surpassed only by fatigue [4,5].

The pathophysiological mechanisms underlying these persistent respiratory symptoms remain unclear [6]. Suggested contributors include structural changes in both pulmonary vasculature and airways, likely triggered by inflammation following Severe Acute Respiratory Syndrome Coronavirus 2 (SARS-CoV-2) infection [7,8]. Vascular mechanisms such as endothelial damage [8], microthrombosis, and macrothrombosis have been reported, along with evidence of angiogenesis [9]. Structural bronchial changes, including organising pneumonia, bronchiolisation of alveoli, and interstitial fibrosis, have also been described [10]. Such findings suggest a complex interplay between vascular and airway changes following initial infection, contributing to impaired pulmonary function. To improve outcomes for patients with long-term effects of COVID-19, it remains crucial to explore how vascular and bronchial sequelae impact lung function, highlighting a critical knowledge gap and the need for precise, non-invasive detection methods.

Functional respiratory imaging (FRI) is an imaging technique that addresses this need by providing detailed assessments of lung structure and function. By combining high-resolution computed tomography (HRCT) data with computational fluid dynamics (CFD), FRI generates three-dimensional maps of airways and vascular structures, providing valuable insights into pulmonary function and airflow dynamics [11]. As its spatial resolution is limited by the underlying CT data, FRI does not directly assess the alveolar-capillary interface where gas exchange occurs, and its relevance for assessing gas exchange impairment is therefore based on indirect measurements. FRI is particularly valuable in cases where traditional methods fail to identify subtle structural changes. It has proven useful in detecting blood and airflow abnormalities associated with various lung diseases, including those seen following SARS-CoV-2 infection [12].

Previous studies have demonstrated FRI’s ability to detect vascular abnormalities during the acute phase of COVID-19, including findings of reduced blood volume in small peripheral vessels and a compensatory increase in larger central arteries in severely ill patients [13]. Similar vessel reduction has been linked to reduced diffusing capacity of the lung for carbon monoxide (DLCO) [14].

Building on previous FRI findings that identified pulmonary changes during the acute phase of COVID-19, this study extends the investigation by examining COVID-19 patients eight months after hospital discharge. This study aims to bridge the current knowledge gap by identifying whether long-term structural and functional changes are present, and detectable using FRI, and by exploring how such changes relate to impaired lung function, including DLCO. Such findings could contribute to improved understanding and targeted management of persistent lung impairment.

## Materials and methods

A retrospective analysis was conducted within the framework of a prospective longitudinal observational study, to evaluate associations between FRI findings extracted from HRCT scans and pulmonary function test (PFT) results, in previously hospitalised adult COVID-19 patients from a single-centre (Karolinska University Hospital, Stockholm, Sweden). Patients were recruited at the dedicated Post-COVID outpatient clinic at Karolinska University hospital, Stockholm, Sweden, beginning 2^nd^ of June 2020. The FRI cohort was stratified from the REDCap database within the ongoing UppCov and ReCov study, for which all participants had provided written informed consent prior to enrolment. Previous research has described the UppCov and ReCov data assessment in detail [15]. The present substudy (ImCov) was approved by the Swedish Ethical Review Authority (application number 2021–04735), which confirmed that no additional consent was required, as the existing consent covered the planned secondary analysis. The first access to data for research purposes was on 04/06/2021. Additional data access and image selection for the ImCov substudy were performed on 08/11/2021. The CT images were subsequently sent to FLUIDDA NV (Belgium), developer of the FRI platform, for FRI analysis on 16/03/2022.

All data were pseudo-anonymised before being made available to the research team. No re-identification was possible at the level of FRI analysis. Ethical approval for the entire study was obtained from the Swedish Ethical Review Authority (Dnr: 2020–02394, 2020–02149, and 2021–04735).

At the time of initial data accessed for research purposes on 04/06/2021, 189 patients had accepted participation in UppCov. From this cohort, a stratified selection of 30 male patients was made on 08/11/2021 based on the following criteria: age 50–70 years, resting oxygen saturation >95%, BMI ≥ 25, and a recorded HRCT scan performed ≥4 months after discharge. Only patients with a positive reverse transcription polymerase chain reaction (RT-PCR) test for SARS-CoV-2 were included. Exclusion criteria were current smoking, a medical history of previous respiratory disease, ECMO treatment during hospitalisation, insufficient image quality on recorded HRCT exams, and non-COVID-related radiological changes that could affect the FRI analysis. These criteria were applied to define a relatively uniform subgroup enriched for common risk factors associated with severe COVID-19, in order to minimise variability that could obscure potential associations between structural and functional lung changes, especially given the limited cohort size.

**Fig 1:**
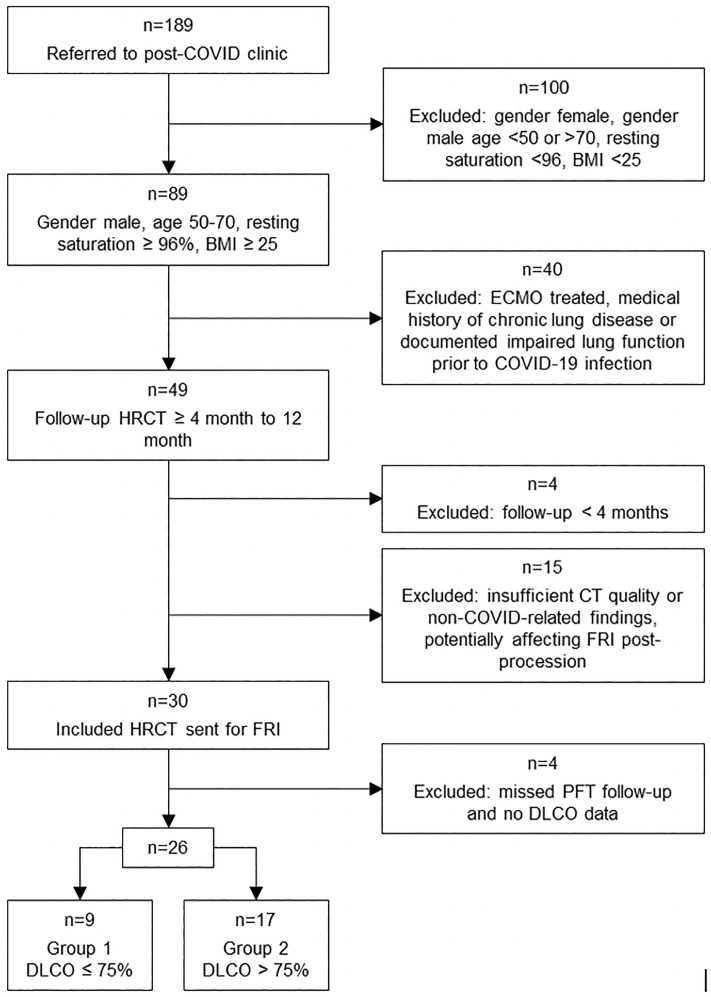
Patient Selection Flowchart. Flowchart of the inclusion process for 26 male post-COVID-19 patients who underwent FRI assessment eight months after hospital discharge.

The follow-up CT scans of the thirty selected patients were sent for post-processing with FRI software on 16/03/2022. Among these, 26 patients met the necessary follow-up criteria, based on available PFT data including DLCO, and were included in the final cohort (Fig 1). The 26 CT scans used for FRI analysis were performed with a median of 8 months after discharge, while the corresponding lung function measurements were conducted at a median of 9 months after discharge. The time interval between the CT scans and PFTs had a median [IQR] of 0.4 [−0.3–3.8] months.

### Imaging

#### CT protocol.

All CT scans were performed using a SOMATOM Definition Flash dual-source CT scanner (2 × 64 slices) (Siemens Healthineers, Germany) with a protocol design of a single-source acquisition. Chest CT scans were acquired for all patients during both inspiratory and expiratory phases. The scans were reconstructed with a slice thickness of 0.6 mm, with no overlap, and used a sharp algorithm (Br59) with first-level iterative reconstruction (f SAFIRE 1) to enhance spatial resolution while reducing noise.

The dose length product ranged between 50 and 337 milli-gray* cm (mGy*cm), with a CT dose index volume of 2–7 mGy. Although the protocol was originally designed for imaging 6 months post-discharge, the actual median time [interquartile range, IQR] to CT was 8 [5–10] months, reflecting variation in clinical scheduling.

#### FRI analysis.

The methods for analysing pulmonary vascular volumes using CT scans have been described in detail in previous research by Lins et al. [16].

In summary, by post-processing CT scans, the functional respiratory imaging technique (FRI; FLUIDDA, NV, Belgium) extracts additional information from the scans. FRI utilises automated tissue segmentation algorithms powered by deep learning to generate quantitative assessments of lung tissue, providing measurements of vessel and bronchial volumes, which leads to more detailed information from the CT scans. Although FRI offers a highly detailed analysis of pulmonary function and morphology, the data are naturally still limited by the resolution of the original CT images.

Pulmonary vasculature volumes (mL) were evaluated in three groups based on vessel cross-sectional areas: blood vessels with a cross-sectional area < 5 mm² (BV5), blood vessels with a cross-sectional area larger than 5 mm² but smaller than 10 mm² (BV5–10), and blood vessels with a cross-sectional area larger than 10 mm² (BV10). These measurements represent the combined volumes of macroscopic intrapulmonary vessels.

In addition to absolute volumes, the proportion (%) of vessel volume relative to the total pulmonary blood volume (TBV) was calculated as BVX (%), providing a standardised measure to compare the relative contribution of vessel sizes between groups.

To facilitate group comparisons of airways, FRI airway measurements were provided to us by the FLUIDDA company as percent predicted (%), using proprietary reference values adjusted for sex, age, weight, and height (no established reference values exist for pulmonary vessel volumes). From these percent predicted values, predicted image-based lobar volumes (iVlobe%) and predicted specific image-based airway volume (siVaw%) were derived. SiVaw % was defined as the ratio of airway volume (iVaw) to lobar volume (iVlobe) and expressed as a percentage of predicted values (%). An increase in siVaw % may reflect airway widening, e.g., bronchial dilatation, as previously undetectable smaller branches become visible on CT [17]. Because siVaw % is a ratio of airway to lobar volume, it may also increase as a result of reduced lobar volume. FRI provides airway volume assessments with resolutions of 0.23–0.35 mm [18,19]. Perfusion measurements (IVQ) were calculated by relating the proportion of blood vessel volume (BVX%) to total pulmonary blood volume (TBV) and weighting this by the percentage of total inspiratory capacity in each lobe.

### Lung function

Pulmonary function tests, including DLCO, were conducted at a median of nine months post-discharge, and in accordance with the guidelines from the American Thoracic Society and European Respiratory Society [20,21].

Static lung volumes included measurements of total lung capacity (TLC), residual volume (RV), and functional residual capacity (FRC). Dynamic spirometry included measurements of forced vital capacity (FVC) and forced expiratory volume in one second (FEV₁) and the FEV₁/FVC ratio. No bronchodilators were administered prior to spirometry. DLCO and KCO (carbon monoxide transfer coefficient; DLCO/VA were measured separately as part of the pulmonary function test. Normal values for each parameter were determined using Swedish reference equations for adult men by Hedenström et al. [22].

Based on the DLCO results, patients were divided into two groups: those with DLCO values ≤ 75% of the predicted value (n = 9) and those with values > 75% (n = 17), adjusted for haemoglobin levels. This threshold of 75% was selected based on commonly accepted clinical guidelines [23] and its established role as an indicator of impaired gas exchange in post-COVID-19 patients [24]. It differentiates patients with significant diffusion impairment from those with relatively preserved pulmonary function, facilitating comparisons of structural changes in pulmonary vasculature and airways. The groups are hereafter referred to as the low DLCO group and normal DLCO group, respectively.

### Statistical analysis

The anthropometric data and FRI parameters are presented as medians with interquartile ranges (IQRs). P-values were calculated using the Mann-Whitney U test to compare groups divided by DLCO values (≤ 75 and > 75). The Mann-Whitney U test was consistently employed for continuous variables, as the small sample size did not allow the assumption of normal distribution of the data. For categorical variables, chi-square tests were used to assess potential differences between the groups.

Regression analyses were conducted to examine the relationship between the independent variables, including the FRI parameters BV5% (proportion of blood vessel) and siVaw (specific image-based airway volume), and the dependent variable DLCO. Additionally, multiple regression analyses were performed to evaluate the combined effects of these variables of interest.

Given the small sample size, the robustness of the regression models was carefully considered, and the standard regression assumptions were confirmed to be met. A p-value of less than 0.05 was considered statistically significant throughout all analyses.

All statistical analyses were conducted using SPSS version 28 (IBM, Armonk, New York, USA).

### Ethical considerations

Our cohort was stratified from a larger COVID-19 follow-up study, in which participants, before inclusion, were given oral and written information about the purpose of the study, and each participant gave their written informed consent. UppCov is an ongoing collaboration between several departments at Karolinska University Hospital and Karolinska Institutet. The principles of the Declaration of Helsinki were followed. The ongoing longitudinal COVID-19 studies (UppCov and ReCov), and the present study (ImCov) were approved by the Swedish Ethical Review Authority (application number 2020–02394, 2020–02149, and 2021–04735).

## Results

### Study population

Baseline characteristics, including demographics and hospitalisation details, are summarised in Table 1. Age and BMI was similar between the groups. While the proportion of patients treated in intensive care did not differ (p = 0.129), the low DLCO group had longer period of care (p = 0.001). Similarly, there was no difference in the proportion of patients requiring mechanical ventilation (p = 0.190), but the low DLCO group required it for a longer duration (p = 0.016). The low DLCO group had more patients with a history of smoking compared to the normal DLCO group (p < 0.001).

**Table 1:** Selected baseline characteristics.

Variable	Full cohort (n = 26)	Group 1 (DLCO ≤ 75) (n = 9)	Group 2 (DLCO > 75) (n = 17)	P-value
Age at admission (years)	60 (54–66)	65 (61-67)	57 (53- 64)	0,051
BMI (kg/m ^2^) at first follow-up	28 (25–30)	29 (25–30)	27 (25–31)	0,634
Hospitalisation duration (days)	29 (13–52)	53 (41–64)	16 (8–35)	<0,001
Intensive care treated (yes/no)	21/5	9/0	12/5	0.129 ^**a**^
Intensive care treated (days)	10 (4–22)	21 (14–35)	7 (0–11)	0,001
Mechanical ventilation (yes/no)	18/8	8/1	10/7	0,190 ^**a**^
Time on mechanical ventilator (days)	8 (0–23)	14 (10–27)	5 (0–9)	0,016
Previous tobacco use (yes/no)	14/12	9/0	5/12	<0,001 ^**a**^
Pack year tobacco (years) *	0 (0–14)	9 (7–23)	0 (0–0)	0,014

Baseline demographic and clinical characteristics of 26 previously hospitalised male COVID-19 patients, assessed approximately eight months post-discharge. Patients were stratified by DLCO: ≤ 75% (n = 9) and >75% (n = 17). Data are shown as median (interquartile range, IQR) or number (%). P-values were calculated using the Mann-Whitney U or Chi-square test as appropriate. *n = 22; two missing values in each group. **Definition of abbreviations:** BMI = body mass index; DLCO = diffusing capacity of the lung for carbon monoxide.

### Key FRI findings in relation to DLCO

#### Blood vessels.

CT-derived FRI measures are summarised in Table 2. Low DLCO was associated with a lower proportion of blood vessels with a diameter of less than 5 mm² (BV < 5%) compared to patients with normal DLCO (median 56% vs. 60%, p = 0.008) (Table 2). Additionally, low DLCO was also linked to a higher percentage of blood vessels between 5 and 10 mm² (BV5–10%) (median 20% vs. 18%, p < 0.001), and larger vessels (BV > 10%) (median 27% vs. 21%, p = 0.021).

**Table 2:** CT-derived FRI measures.

Variable CT-derived FRI measures	Full cohort (n = 26)	Group 1 (DLCO ≤ 75) (n = 9)	Group 2 (DLCO > 75) (n = 17)	P-value
**Blood vessels (total lung region)**				
BV5 (%)	59 (55–63)	56 (51–59)	60 (56–64)	0,008
BV5–10 (%)	19 (17–20)	20 (19–21)	18 (17–19)	<0,001
BV10 (%)	22 (19–27)	27 (21–29)	21 (18–25)	0,021
BV5 (ml)	140 (126–164)	129 (121–151)	155 (132–175)	0,025
BV5–10 (ml)	47 (41–51)	49 (44–54)	45 (39–50)	0,181
BV10 (ml)	53 (45–72)	62 (46–75)	49 (44–67)	0,241
**Airway (total lung region)**				
Image-based airway volume (ml) **iVaw** *	68 (61–81)	74 (63–84)	66 (60–80)	0,263
Image-based airway volume percent predicted **iVaw %** *	106 (97–131)	122 (98–136)	106 (96–124)	0,440
Image-based Lobar volumes percent predicted iVLobe % *	81 (77–93)	72 (65–81)	90 (79–95)	0,001
Specific image-based airway volume percent predicted siVaw % *	131 (101–144)	149 (138–165)	117 (93–132)	0,002
Ventilation/Perfusion IVQ	14 (11-15)	11 (10–14)	14 (12–16)	0,034

Quantitative CT-derived measures of pulmonary vasculature and airway structure in the same cohort. Assessed with functional respiratory imaging (FRI) at approximately eight months post-discharge. Groups defined by DLCO ≤75% and >75%. Data are presented as median (IQR). P-values calculated with the Mann-Whitney U test. *n = 25; one missing value in Group 1. **Definition of abbreviations:** DLCO = diffusing capacity of the lung for carbon monoxide; CT = computed tomography; FRI = Functional respiratory imaging; BV5 (%), BV5–10 (%), BV10 (%) = pulmonary blood vessel volumes by size category, representing the blood vessel volume with a cross-sectional area <5 mm², 5–10 mm², and >10 mm² as a percentage of total blood vessel volume; BV5, BV5–10, BV10 = pulmonary blood vessel volumes by size category, representing the blood vessel volume with a cross-sectional area <5 mm², 5–10 mm², and >10 mm², respectively, expressed in millilitres (mL); iVaw = image-based airway volume, expressed in millilitres (mL), measured at inspiration (TLC) in the total lung region; iVaw % = image-based airway volume as percent predicted, measured at inspiration; iVLobe % = image-based lobar volume predicted; expressed as a percentage of predicted values, measured at inspiration, here representing the total lung region, i.e., total lung volume; siVaw % = specific image-based airway volume predicted; expressed as the ratio of airway volume to lobar volume (iVLobe), expressed as a percentage of predicted values, measured at inspiration; IVQ = ventilation/perfusion ratio, measured at expiration/inspiration.

Similarly, low DLCO was associated with a lower absolute volume of the smallest blood vessels (BV < 5) compared to normal DLCO (median 129 ml vs. 155 ml, p = 0.025). In contrast, no differences were observed in the absolute volumes of larger vessels (BV5–10 and BV > 10) between the two groups (p = 0.181 and p = 0.241, respectively).

#### Airways.

In patients with low DLCO, the specific image-based airway volume (siVaw), was higher compared to those with normal DLCO (median 149% vs 117%) (p = 0.002).

Across the entire cohort, the image-based airway volume exceeded the predicted reference value of 100%, indicating generally elevated airway volumes relative to lobar volumes in the study population.

The absolute airway volume (iVaw, mL) was slightly higher in the low DLCO group (median 74 ml) than in the normal DLCO group (66 ml), although this difference was not statistically significant (p = 0.263).

#### Lung volumes.

Lobar volumes (iVlobe %) were reduced in both groups, with the low DLCO group showing a more pronounced reduction compared to the normal DLCO group. The median iVlobe % in the low DLCO group was 72%, while in the normal DLCO group, it was 90% (p = 0.001), suggesting a decrease in total lung volume, particularly in the low DLCO group.

#### Correlation and regression analyses between FRI findings and DLCO.

**Fig 2:**
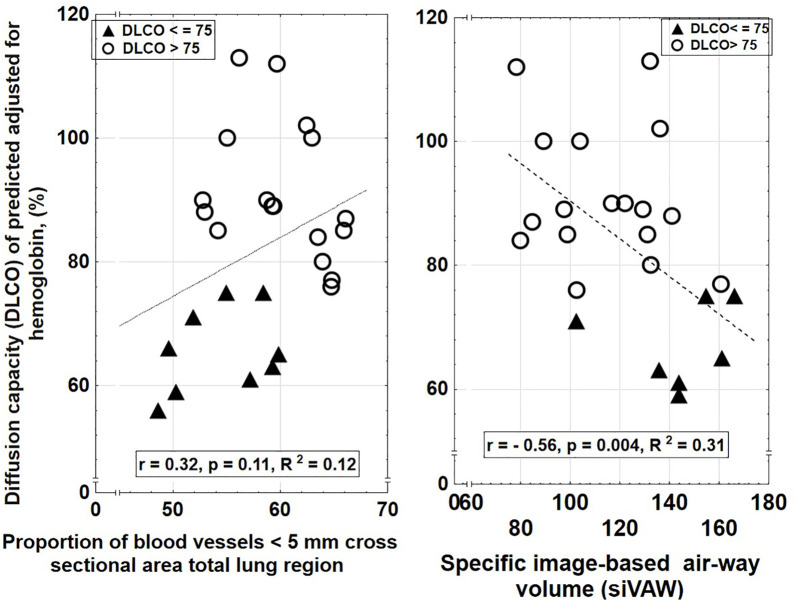
Relationship between DLCO and FRI-derived structural lung changes. The relationship between DLCO and FRI-derived lung structure changes in 26 previously hospitalised covid-19 patients, eight months post discharge. Filled triangles represent subjects with DLCO ≤ 75%, and unfilled circles represent subjects with DLCO > 75%. The left panel illustrates a positive trend between DLCO and the proportion of small pulmonary blood vessels (BV5%), while the right panel depicts an inverse correlation between DLCO and predicted specific airway volume (siVaw). This relationship is described by the function Y = 26.75 + 0.95* X (r = 0.32, p = 0.11, R² = 0.10) and Y = 119.87 - 0.295 * X (r = – 0.56, p = 0.004, R² = 0.31) respectively.

To further investigate the relationship between structural lung changes and lung function, linear correlation and regression analyses were performed between FRI-derived parameters and DLCO values. In regression analysis, no significant correlation was observed between BV5% and DLCO (r = 0.32, R² = 0.103, p = 0.11) (Fig 2), indicating that changes in the proportion of small blood vessels were not significantly associated with variations in DLCO. In contrast, a moderate inverse correlation was found between siVaw and DLCO (r = – 0.56, R² = 0.310, p = 0.004) (Fig 2), suggesting that as specific airway volume increases, DLCO tends to decrease.

When both BV5% and siVaw were included in a multiple regression model, siVaw remained statistically significant ($\hat{\beta }\;$= – 0.269, p = 0.012), while BV5% did not ($\hat{\beta }$ = 0.475, p = 0.392). The overall model was statistically significant (p = 0.014), with an adjusted R² of 0.258.

### DLCO and lung volumes

#### Pulmonary function.

Pulmonary function tests parameters are summarised in Table 3. DLCO values were used to categorise the two groups, as previously described, with the low DLCO group having median [IQR] DLCO values of 65% [IQR 60–73], compared to 89% [IQR 85–100] in the normal DLCO group (p < 0.001) (Table 3). This categorisation forms the basis for the analyses presented in this study.

**Table 3:** Pulmonary function tests.

Variable	Full cohort (n = 26)	Group 1 (DLCO ≤ 75) (n = 9)	Group 2 (DLCO > 75) (n = 17)	P-value
**PFT**				
DLCO (corr. Hb, % predicted)	85 (70–90)	65 (60–73)	89 (85–100)	
FEV1 (% predicted)	91 (83–98)	86 (78–93)	93 (85–99)	0,133
FVC (% predicted)	87 (82–98)	81 (70–87)	93 (85–99)	0,021
FEV1/FVC (ratio)	0,78 (0,72–0,83)	0,83 (0,76–0,87)	0,76 (0,70–0,82)	0,051
FEV1/FVC (% predicted) *	106 (100–115)	110 (103–118)	105 (95–113)	0,074
VC (% predicted) **	88 (82–96)	83 (68–88)	91 (83–97)	0,057
TLC (% predicted)	90 (75–94)	73 (64–85)	92 (85–98)	0,003
FRC (% predicted)	85 (78–105)	77 (62–87)	95 (82–109)	0,013
RV (% predicted)	91 (75–108)	77 (47–109)	94 (80–107)	0,287
KCO (DLCOc/VA Pre)	1.29 (1.14–1.48)	1.19 (1.02–1.38)	1.33 (1.21–1.53)	0.075
KCOc (DLCOc/VA % Ref)	95 (86–107)	92 (75–101)	96 (92–113)	0.200

Key pulmonary function test (PFT) parameters in the study cohort assessed at follow-up, including DLCO, lung volumes, transfer coefficient (KCO) and spirometry values. Grouping based on DLCO ≤75% vs > 75%. Results are presented as median (IQR). *n = 25; one missing value in Group 2; **n = 25; one missing value in Group 1. **Definition of abbreviations**: DLCO = diffusing capacity of the lungs for carbon monoxide; corr. Hb = corrected for haemoglobin; % predicted = percent predicted, expressed as a percentage of the predicted value; FEV1 = forced expiratory volume in 1 second; FVC = forced vital capacity, the total volume of air exhaled during a maximally forced breath out; FEV1/FVC = ratio of forced expiratory volume in 1 second to forced vital capacity; VC = vital capacity, the air volume exhaled after the deepest inhalation; TLC = total lung capacity, the volume in the lungs at maximal inflation (the sum of VC and RV); FRC = functional residual capacity, the volume in the lungs at the end-expiratory position; RV = residual volume, the volume of air remaining in the lungs after a maximal exhalation; KCO = transfer coefficient for carbon monoxide (DLCO divided by alveolar volume), here shown as both absolute values (Pre) and percent predicted (% Ref); DLCOc/VA = DLCO corrected for haemoglobin and adjusted for alveolar volume.

A reduction in total lung capacity (TLC) was observed, with lower values in the low DLCO group compared to the normal DLCO group (median 72.5% [63.5–84.5] vs. 92.0% [85.0–94.5], p = 0.003), consistent with a restrictive ventilatory pattern.

In a simple regression analysis, TLC % predicted explained 32.5% of DLCO % predicted variance (R² = 0.325, p = 0.002).

The FEV₁/FVC ratio was higher in the low DLCO group (median 0.83, IQR 0.76–0.87) compared to the normal DLCO group (median 0.76, IQR 0.70–0.82), indicating a similar restrictive pattern, though the difference was not statistically significant (p = 0.051).

KCO (DLCOc/VA), representing gas transfer per unit alveolar volume, expressed in mmol/(min·kPa·l), was also lower in the low DLCO group (median 1.19) compared to the normal DLCO group (median 1.33), although the difference was not statistically significant (p = 0.075).

#### Physiological lung volume versus anatomical (FRI) estimates.

TLC % predicted, was strongly associated with total iVlobe (% predicted), the anatomically derived FRI estimate of lung volume (R² = 0.65, p < 0.001). IVlobe and TLC also showed a moderate to strong correlation (Spearman r = 0.83, p < 0.001).

## Discussion

Building on prior findings of structural changes in pulmonary vessels and airways during the acute phase of COVID-19 [12–14], we demonstrate that such changes are present and are associated with impaired gas exchange even eight months after discharge. Our findings of redistribution of pulmonary blood flow from the smallest vessels, together with airway enlargement, suggest ongoing vascular and bronchial involvement in individuals with prior COVID-19 and gas exchange disturbances. These findings support the hypothesis that COVID-19-induced inflammation contributes to both small pulmonary vessel loss and bronchial dilatation, which impair lung function over time.

Similar impairments in DLCO and total lung capacity several months after COVID-19 have also been reported by Pini et al. (2023), particularly among patients previously treated in intensive care. In their study, a combined diffusion and restrictive deficit was more frequently observed in ICU survivors, and more than 60% of these patients showed impaired lung function 4–6 months post-discharge [25]. These findings are consistent with those observed in our cohort. Although baseline values were not available in either study, these observations support the notion that measurable gas exchange limitation may be present at follow-up.

Our findings of vascular redistribution are consistent with those reported during the acute phase of COVID-19 [13] and at 2–3 months post-discharge [14], where impaired DLCO was linked to a shift in blood flow from the periphery to larger central vessels. Notably, this pattern appears unique to COVID-19, as it was not observed in matched ARDS patients of non-COVID origins, albeit in a small cohort [13]. FRI-derived vessel measurements have been validated against histological findings, confirming that reduced peripheral blood volume corresponds to small vessel loss [26].

The reduction of small vessel volumes observed in this study underpins the hypothesis that small vessel loss is a contributor to impaired gas exchange. The peripheral distribution of inflammation and signs of structural damage, consistently reported in acute and follow-up studies of COVID-19 [27,28], further highlights the susceptibility of these vessels to injury, likely explaining their reduction in both absolute volume and proportion. However, despite significant group differences in small vessels, this parameter did not significantly correlate with DLCO in regression analysis. One possible explanation is that pulmonary vascular remodelling in post-COVID lungs may be spatially heterogeneous and not fully captured by global BV5% measurements. Alternatively, loss of small vessels may not be the primary limiting factor for diffusing capacity in this population, especially in the context of co-existing volume restriction.

Vessel loss aligns with impaired diffusion capacity and the hypothesis of peripheral endothelial injury as a key driver of post-COVID-19 dysfunction. Endothelial injury is a hallmark of COVID-19-induced pulmonary dysfunction, with inflammation and immune-mediated damage targeting the pulmonary microvasculature [8]. Autopsy studies have shown pulmonary endothelitis [29], microvascular distortion, enhanced bronchial circulation, and increased angiogenesis during the acute phase of COVID-19 [9]. Selective loss of small vessels, alongside compensatory intussusceptive angiogenesis, has been identified as a hallmark of COVID-19 pathology, distorting vascular architecture without restoring function [9]. These processes may explain the vascular changes observed in this study, suggesting long-term alterations in pulmonary circulation.

Airway remodelling also plays a critical role. SiVaw, calculated as a ratio of airway volume to lobar volume, provides a standardised measure of airway dilatation relative to lung size [30]. The increased siVaw observed in patients in the low DLCO group is interpreted as bronchial dilatation secondary to parenchymal contraction and traction, in line with fibrotic remodelling processes. This finding, supported by the inverse correlation between siVaw and DLCO, suggests that airway dilatation contributes to impaired gas exchange. This interpretation should be balanced against the lung volume data, which support a restrictive pattern, and the observation that the elevated siVaw is also driven by reduced lobar volumes, rather than only by an increase in airway volume. While the exact mechanisms remain unclear, structural airway changes, including bronchial dilation and fibrosis, have been observed on CT up to a year post-infection [31]. Persistent dilation, as seen in this study, likely reflects pathological airway remodelling, disrupting normal elastic recoil and gas exchange efficiency.

Histopathological findings support these observations. A study of autopsies from 95 patients conducted an average of four months post-COVID-19, identified pathological bronchial wall changes, further implicating airway involvement in post-COVID-19 dysfunction [32]. Our multiple regression analyses revealed that siVaw explained 31% of the variation in DLCO, compared to 10% explained by BV5%, underscoring the impact of bronchial remodelling on diffusion impairment.

This study supports our hypothesis of fibrotic changes, as evidenced by reduced lung volumes and a restrictive ventilatory pattern observed in both pulmonary function test and FRI. In this context, the observation that KCO values were slightly lower in the low DLCO group, although still within the normal range, may indicate a reduction in effective alveolar surface area, supporting the interpretation of mild to moderate parenchymal involvement rather than isolated vascular pathology. While both TLC and iVlobe metrics reflect total lung volume, TLC is a physiologically derived parameter that may be influenced by non-ventilated or poorly ventilated lung regions, whereas iVlobe represents a CT-derived anatomical measurement. The strong correlation between TLC and iVlobe found in our study supports the role of iVlobe as an imaging-based approximation of physiologically measured total lung capacity.

Patients in the low DLCO group exhibited significantly lower total lung capacity (TLC) and lobar volumes (iVlobe%), while elevated FEV ₁/FVC ratios further reinforce this restrictive pattern. These findings align with previous reports of persistent fibrosis-like abnormalities on CT at six months and beyond [33,34]. Similarly, our earlier research identified fine reticular patterns in 93% of ICU-treated patients two years post-discharge, with higher opacity scores correlating with reduced DLCO [35].

The reductions in TLC and iVlobe%, coupled with elevated FEV₁/FVC ratios, underscore the role of fibrotic processes in post-COVID-19 lung dysfunction. Clinically, these findings underscore the importance of assessing DLCO in post-COVID-19 evaluations, as impaired values may indicate underlying structural airway and vascular changes. Future research should validate these results in larger cohorts, investigate the mechanisms driving airway dilatation, and determine whether these changes represent permanent scarring or ongoing remodelling with potential for therapeutic intervention.

### Strengths and Limitations

One notable strength of this study is the use of Functional Respiratory Imaging (FRI), which enabled detailed, lobe-specific quantification of both airway and vascular structures, features not easily captured with conventional imaging methods. By correlating these image-derived parameters with DLCO, a clinically established measure of pulmonary function, we enhance the translational relevance of our findings.

Further strengths include the clearly defined, homogeneous cohort of hospitalised male patients with similar BMI and age ranges, reducing confounding and increasing internal validity. As all patients were consecutively included early in the first pandemic wave, none were vaccinated, allowing assessment of the untreated natural course of COVID-19. The relatively uniform follow-up time point (eight months post-discharge) adds temporal consistency. Moreover, the structured follow-up programme after discharge, likely reduced selection bias and loss to follow-up, particularly among patients with more severe disease or persistent symptoms. As such, the cohort may more accurately reflect the spectrum of post-COVID-19 lung involvement among hospitalised individuals. Additionally, the finding that bronchial dilation was more strongly associated with reduced DLCO than the reduction in small pulmonary vessels offers novel insight into the pathophysiology of post-COVID-19 lung dysfunction.

Nevertheless, this study has several limitations. The small sample size (n = 26) and demographic homogeneity restrict statistical power and generalisability to broader populations. While strict inclusion criteria reduce confounding, they also limit external validity. The absence of a healthy control group further constrains interpretation, and the use of a single-centre study design may limit the generalisability of the findings. Although relative comparisons were enabled through predicted values for siVaw, no normative data exist for vascular metrics such as BV5%, which necessitates cautious interpretation of percentage-based distributions. Moreover, the use of a single imaging modality (FRI) may not capture the full complexity of pulmonary changes in COVID-19 patients. Additionally, DLCO z-scores (the standard deviations from mean average for healthy individuals) were only available as visual indicators and not as numerical outputs, which introduced a minor risk of misclassification in a few borderline cases. Lastly, the study’s findings are based on a relatively short follow-up period (eight months), and the cross-sectional design precludes any assessment of temporal changes, making it unclear whether the observed structural changes are progressive, permanent or reversible.

## Conclusion

This study demonstrates that structural lung changes are present in previously hospitalised COVID-19 patients, eight months post-discharge. These alterations, quantified using functional respiratory imaging (FRI), primarily affected small airways and pulmonary vessels. Notably, siVaw (i.e., the ratio of airway volume to lobar volume) was more strongly associated with reduced DLCO than the proportion of small vessels. While this suggests that airway remodelling may play a prominent role in post-COVID-19 lung dysfunction, our findings point to a mixed picture in which multiple mechanisms, including vascular and parenchymal factors, likely contribute. Clinically, our findings underscore the importance of monitoring patients with impaired DLCO for airway, parenchymal and vascular abnormalities. Further studies, including histopathological validation, are needed to clarify the underlying mechanisms and inform targeted interventions.

## Supporting information

S1 File FRI supplementary data 1. (PDF)

S1 Check list PLOSOne Human Subjects Research Checklist. (DOCX)
