# Supplementary material for: Pulmonary vascular and airway changes in previously hospitalised COVID-19 patients: Long-term functional respiratory imaging findings correlate with reduced DLCO
Source: PLoS One. 2025 Dec 2;20(12):e0335075. doi: 10.1371/journal.pone.0335075 (PMC12671730; doi:10.1371/journal.pone.0335075)
Supplement: S1 File — (PDF) [file pone.0335075.s001.pdf]

	BV5TLC0TotalLun	BV5PRTLC0Tota	BV510TLC0TotalLu	BV510PRTLC0Total	BV10TLC0	BV10PRTL
1	178,65	54,22	56,68	17,20	94,19	28,58
2	120,52	50,24	49,61	20,68	69,77	29,08
3	118,82	59,24	40,19	20,04	41,57	20,72
4	130,83	62,50	40,64	19,42	37,84	18,08
5	164,69	63,52	47,47	18,31	47,10	18,17
6	159,56	63,96	41,82	16,77	48,08	19,27
7	176,97	66,12	45,08	16,84	45,61	17,04
8	172,87	52,99	64,31	19,71	89,07	27,30
9	163,59	58,77	48,91	17,57	65,84	23,65
10	134,55	57,18	46,35	19,70	54,40	23,12
11	180,29	64,78	44,58	16,02	53,43	19,20
12	186,77	65,94	47,00	16,59	49,46	17,46
13	137,08	64,80	37,03	17,50	37,44	17,70
14	133,77	62,94	35,75	16,82	43,03	20,24
15	126,84	59,70	38,19	17,97	47,42	22,32
16	127,97	49,59	52,99	20,54	77,07	29,87
17	123,33	58,43	42,87	20,31	44,87	21,26
18	154,55	59,42	50,30	19,34	55,24	21,24
19	140,99	52,82	51,94	19,45	74,02	27,73
20	154,02	54,96	51,38	18,34	74,84	26,70
21	143,48	59,84	47,76	19,92	48,54	20,24
22	111,38	59,30	36,89	19,64	39,56	21,06
23	125,80	55,06	44,28	19,38	58,40	25,56
24	153,36	51,88	57,10	19,31	85,17	28,81
25	151,11	56,17	49,77	18,50	68,12	25,32
26	123,31	48,58	55,10	21,71	75,44	29,72

IVAWTLC0	IVAWPPTLC	IVLOBETLC	IVLOBEPPTL	IVQFRCTLC	SIVAWTLC	SIVAWPPTI	CTmonths_
62,20	97,67	6,42	88,82	10,23	9,70	98,95	5
59,67	96,63	4,24	61,37	9,16	14,09	143,74	11
64,40	100,34	4,84	66,35	14,28	13,29	135,64	8
60,35	100,82	4,52	68,65	12,05	13,35	136,26	8
57,39	93,68	7,33	107,37	16,46	7,84	79,95	11
81,94	118,28	6,31	77,89	15,17	12,99	132,51	8
58,77	89,49	7,08	93,87	14,17	8,30	84,72	4
111,82	156,62	8,09	96,10	15,61	13,82	141,02	9
71,86	110,26	6,01	80,57	10,63	11,95	121,98	7
84,61	133,88	6,01	84,09	13,68	14,09	143,76	10
72,88	106,02	7,26	90,52	14,31	10,04	102,44	7
71,26	115,41	5,55	80,38	10,85	12,84	131,07	11
80,28	136,23	5,10	79,37	14,02	15,75	160,69	9
48,88	77,34	5,59	78,22	12,82	8,75	89,28	7
59,14	94,30	7,70	109,08	20,16	7,68	78,33	9
74,94		4,40		10,16	17,04		4
68,36	115,10	4,20	64,52	10,90	16,29	166,25	8
95,61	145,58	7,54	100,00	15,16	12,68	129,36	10
66,29	103,28	5,79	79,30	13,28	11,45	116,81	9
79,75	129,15	5,26	76,21	11,25	15,16	154,71	4
85,70	137,72	5,43	77,83	15,92	15,77	160,94	10
63,16	99,94	6,61	92,52	21,46	9,56	97,54	11
63,90	105,92	6,28	94,19	16,53	10,18	103,89	5
63,17	94,73	6,30	81,76	12,52	10,03	102,37	8
79,56	129,86	6,14	89,99	14,67	12,96	132,25	4
84,11	137,28	4,57	67,03	5,02	18,39	187,69	5

demo_sex	tobacco_use	Pack_Year	tot_Hospital_lenght_c
M	0	0	6
M	1		61
M	1	15	53
M	0	0	31
M	0	0	11
M	1		14
M	0	0	9
M	0	0	54
M	0	0	6
M	1		21
M	0	0	1
M	1	32	22
M	0	0	13
M	0	0	7
M	0	0	21
M	1	30	50
M	1	8	31
M	1	31	44
M	0	0	38
M	1	22,5	66
M	1	2	74
M	0	0	40
M	1		26
M	1	9,25	58
M	1	14	16
M	1	7,2	51

intensive_care_y_n	intensive_care_days	mechanical_ventilation_y (1) n (0)
0	0	0
1	34	1
1	21	1
1	10	1
1	5	1
0	0	0
0	0	0
1	30	1
0	0	0
1	8	0
1	8	1
1	1	0
1	5	1
0	0	0
1	11	1
1	12	1
1	15	1
1	16	1
1	29	1
1	35	1
1	55	1
1	10	1
1	10	1
1	26	1
1	7	0
1	20	1

totTIMEINVENTILATOR (days)	BMI_1	DLCOc/VA_KCO Pre	%Ref DLCOc/VA_KCO % I
0	27,8	1,27	93
24	35,9	1,5	106
9	24,4	1,26	96
9	27,4	1,84	127
5	31,2	1,1	78
0	27,7	1,42	104
0	26,5	1,32	92
30	27,4	1,34	95
0	24,6	1,19	91
0	29,0	0,92	72
7	27,4	1,05	75
0	31,3	1,3	94
4	25,0	1,23	96
0	25,9	1,58	121
8	24,7	1,4	100
10	29,4	1,19	92
14	25,1	1,6	122
24	26,4	1,08	82
28	31,5	1,73	121
30	25,1	1,24	94
53	29,1	1,15	87
8	24,9	1,33	96
7	30,4	1,47	109
23	30,1	1	71
0	31,7	1,72	117
10	30,7	1,03	78

group DLCO75	month after discharge_spiro	month after discharge_CT	month diff CT_spiro
2		4,6	
1	9,5	9,8	−,23
1	8,1	7,7	0,46
2	10,7	8,2	2,47
2	9,1	4,2	4,9
2	13,7	7,8	5,88
2	9,7	3,4	6,35
2	9,4	9,8	−,36
2	9,2	8,0	1,18
1	9,5	9,5	0
2	11,9	6,9	4,96
2	10,3	6,1	4,24
2	12,9	9,9	2,99
2	5,6	7,2	−1,61
2	8,1	8,5	−,36
1	8,0	4,0	4,08
1	9,1	9,1	0
2	8,3	9,7	−1,35
2	8,5	8,3	0,16
1	7,8	11,5	−3,72
1	8,3	9,6	−1,38
2	5,0	8,0	−2,99
2	7,4	4,4	2,96
1	9,0	8,7	0,33
2	9,5	4,0	5,46
1	6,9	4,6	2,3

days diff	CT_spiro	pft_fvc_2	pft_fvc_per	pft_fev1_2	pft_fev1_pe	pft_fev1_fv	pft_fev1_fv	pft_vc_perc
		4,5	90	3,5	92	0,778	105	90
−7,00		2,6	52	2,3	60	0,885	116	49
	14	3,5	73	2,8	77	0,800	110	71
	75	4,1	85	3,6	99	0,878	117	83
	149	5,2	109	3,4	93	0,654	87	108
	179	4,7	82	3,9	91	0,830	114	79
	193	4,8	84	3,3	75	0,688	92	85
−11,00		5,68	88	4,72	98	0,831	115	88
	36	4,8	97	3,7	101	0,771	106	97
	0	3,8	85	2,8	86	0,737	106	86
	151	5,8	98	4,1	93	0,707	99	96
	129	4,04	83	3,07	83	0,760		81
	91	3,8	98	2,9	99	0,763	105	95
−49,00		4	85	2,9	83	0,725	100	83
−11,00		5,2	106	3,2	87	0,615	84	107
	124	3,6	81	3	90	0,833	115	82
	0	2,9	68	2,5	79	0,862	120	67
−41,00		5,3	102	3,9	103	0,736	104	104
	5	3,9	69	3,4	79	0,872	116	70
−113,00		4,1	86	3,2	91	0,780	109	89
−42,00		4,1	88	3,5	101	0,854	119	83
−91,00		5,07	99	3,47	90	0,684	93	91
	90	4,3	95	3,4	100	0,791	108	94
	10	5,6	101	3,9	95	0,696	95	99
	166	4,58	93	3,71	98	0,810	106	93
	70	3,3	72	2,9	86	0,879	100	

pft_tlc_perc	pft_frc_perc_ref_2	pft_rv_perc_ref_2	pft_dlco_hb_perc_ref_2
92	104	95	85
61	81	142	59
66	70	59	63
89	112	99	102
112	132	118	84
81	101	87	80
95	95	111	87
93	118	103	88
92	64	85	90
91	84	108	61
90	93	70	76
102	117	154	85
83	82	69	77
82	84	83	100
106	105	101	112
78	90	77	66
57	41	40	75
94	81	76	89
75	79	83	90
74	69	47	75
71	55	47	65
87	78	77	89
94	85	94	100
103	106	109	71
101	106	116	113
73	77	100	56
